# PA1b Inhibitor Binding to Subunits *c* and *e* of the Vacuolar ATPase Reveals Its Insecticidal Mechanism*[Fn FN2]

**DOI:** 10.1074/jbc.M113.541250

**Published:** 2014-05-02

**Authors:** Stephen P. Muench, Shaun Rawson, Vanessa Eyraud, Agnès F. Delmas, Pedro Da Silva, Clair Phillips, John Trinick, Michael A. Harrison, Frédéric Gressent, Markus Huss

**Affiliations:** From the ‡ School of Biomedical Sciences, Faculty of Biological Sciences, University of Leeds, LS2 9JT Leeds, West Yorkshire, United Kingdom,; § Institut National de la Recherche Agronomique, Institut National des Sciences Appliquées-Lyon, Université de Lyon, IFR 41, UMR203 BF2I, Biologie Fonctionnelle Insectes et Interactions, Batiment Louis-Pasteur 20, avenue Albert Einstein, F-69621 Villeurbanne, France,; the ¶ Centre de Biophysique Moléculaire, Centre National de la Recherche Scientifique Unité Propre de Recherche 4301, Rue Charles Sadron, 45071 Orléans cedex 2, France,; the ‖ School of Molecular and Cellular Biology, Faculty of Biological Sciences, University of Leeds, LS2 9JT Leeds, West Yorkshire, United Kingdom, and; ** Abteilung Tierphysiologie, Fachbereich Biologie/Chemie Universität Osnabrück, 49069 Osnabrück, Germany

**Keywords:** ATPase, Drug Action, Electron Microscopy (EM), Membrane Protein, Vacuolar ATPase, PA1b

## Abstract

The vacuolar ATPase (V-ATPase) is a 1MDa transmembrane proton pump that operates via a rotary mechanism fuelled by ATP. Essential for eukaryotic cell homeostasis, it plays central roles in bone remodeling and tumor invasiveness, making it a key therapeutic target. Its importance in arthropod physiology also makes it a promising pesticide target. The major challenge in designing lead compounds against the V-ATPase is its ubiquitous nature, such that any therapeutic must be capable of targeting particular isoforms. Here, we have characterized the binding site on the V-ATPase of pea albumin 1b (PA1b), a small cystine knot protein that shows exquisitely selective inhibition of insect V-ATPases. Electron microscopy shows that PA1b binding occurs across a range of equivalent sites on the *c* ring of the membrane domain. In the presence of Mg·ATP, PA1b localizes to a single site, distant from subunit *a,* which is predicted to be the interface for other inhibitors. Photoaffinity labeling studies show radiolabeling of subunits *c* and *e*. In addition, weevil resistance to PA1b is correlated with bafilomycin resistance, caused by mutation of subunit *c*. The data indicate a binding site to which both subunits *c* and *e* contribute and inhibition that involves locking the *c* ring rotor to a static subunit *e* and not subunit *a*. This has implications for understanding the V-ATPase mechanism and that of inhibitors with therapeutic or pesticidal potential. It also provides the first evidence for the position of subunit *e* within the complex.

## Teaser

**Background:** Pea albumin 1b (PA1b) is a potent and selective inhibitor of insect vacuolar ATPase, but its mechanism is poorly understood.

**Results:** PA1b binds the extracellular surface of the *c* ring and contacts subunit *e*.

**Conclusion:** PA1b inhibits V-ATPase by blocking transition of its rotor past subunit *e*.

**Significance:** This reveals insights into the mechanics and mode of inhibition of the V-ATPase.

## Introduction

The vacuolar H^+^-ATPase (V-ATPase)^4^ is a complex molecular machine responsible for the transmembrane movement of protons against a gradient, fuelled by ATP. It plays a central role in the physiology of virtually all eukaryotic cells, performing such critical functions as acidification of endosomal compartments and energization of secondary active transport (1, 2). The importance of the V-ATPase is also highlighted by the roles it plays in human diseases, such as renal tubular acidosis, inherited forms of deafness, osteopetrosis, autophagic myopathy, pulmonary tuberculosis, and tumor cell survival and invasion (3–6). The V-ATPase has bi-domain architecture similar to that of the F-ATPase (ATP synthase) of mitochondria, consisting of a rotary motor mechanically coupled to a transmembrane proton pump (7, 8). The V-ATPase is, however, substantially larger and more complex, with several unique subunits proposed to be involved in its regulation (1, 9).

**FIGURE 1.**
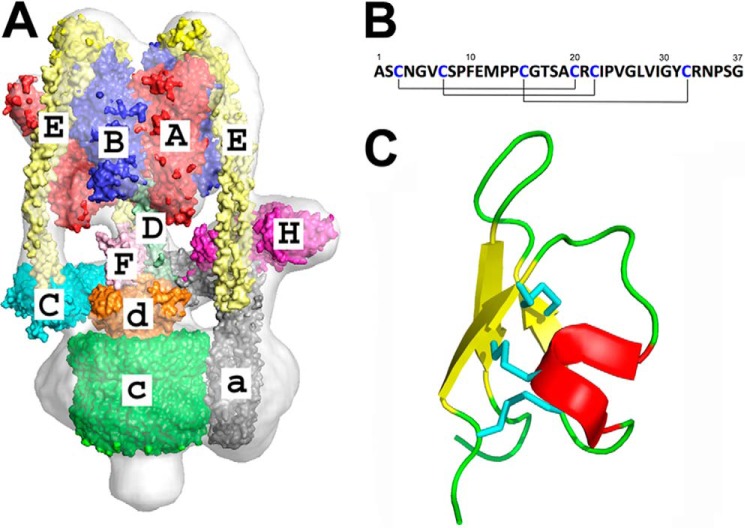
**Organization of the V-ATPase and structure of PA1b.** *A*, the reconstruction *of the M. sexta* V-ATPase from cryo-EM data (11) with crystal structures of homologous subunits fitted and labeled. *B*, primary structure of the insecticidal peptide PA1b. *Lines* indicates the connectivity of the disulfide bridges. *C*, three-dimensional structure of PA1b (Protein Data Bank ID code 1P8B) (20) showing α-helical (*red*), β-sheet (*yellow*), and random coil (*green* regions). Disulfides are colored *cyan*.

Eukaryotic V-ATPase contains at least 29 polypeptides of 13 types. These form a soluble V_1_ domain that hydrolyzes ATP and a V_o_ membrane domain that pumps protons. V_1_ contains subunits A–H with stoichiometry A_3_B_3_CDEF_3_G_3_H, with the ATPase motor consisting of an A_3_B_3_ pseudo-hexamer (10, 11) (Fig. 1*A*). V_o_ is smaller and has subunits *a*, *d*, and *e* and a decameric ring of *c* subunits. Subunits C–H form a network of stalks linking V_o_ to the AB hexamer in V_1_ that function as a stator holding the transmembrane *a* subunit fixed relative to the D–F-*d-c* ring rotor, with this interaction driving proton translocation via a process that remains to be fully resolved.

The roles that the V-ATPases play in bone demineralization and tumor cell survival have made it an important therapeutic target (12, 13). A number of potent inhibitors have been shown to bind tightly to the *c* ring (14–16), presumably preventing proton translocation by obstructing procession of the rotor through the *a* subunit interface. The ubiquity of the V-ATPase has made drug development challenging, but a potential solution is to target different subunit isoforms that are particularly highly expressed in certain cell types. However, a lack of high resolution structural information detailing isoform differences has limited design of targeted inhibitors.

The insecticidal plant toxin pea albumin 1 subunit *b* (PA1b) has been isolated from pea seeds (17–19) and its structure solved (20). This revealed a cystine knot fold with three disulfide bridges and a high degree of stability (Fig. 1, *B* and *C*). PA1b is one of the few orally active entomotoxin peptides currently known and has been shown to be a selective insecticide, acting on numerous agricultural pests and displaying a high toxicity for mosquitoes (17, 19). It has attractive industrial attributes, being extracted from a common agricultural crop (*Pisum sativum*), consumed by humans and other mammals without any reports of toxicity or allergenicity and is suitable for production in transgenic plants. Thus, PA1b could be an attractive alternative to chemical pesticides and could even qualify for use in organic farming. In the rice weevil (*Sitophilus oryzae*) there are strains totally resistant to PA1b (21, 22), hence development of resistance is a potential problem. PA1b has been shown to interact with the V_o_ domain of V-ATPase (23). However, the subunit(s) to which it binds are not known. The basis for its high degree of selectivity and ability to inhibit the V-ATPase from the extracellular environment of the insect gut remain unclear.

Current models of inhibition by other compounds, such as the macrolide bafilomycin, suggest that it is not binding to subunit *c* (or *a*) *per se* that inhibits the enzyme. Rather, inhibition is only expressed when the *c* ring rotates to bring the inhibitor-bound *c* subunit into contact with subunit *a*, hence the apparent additional role for this subunit in bafilomycin (24) and apicularen (14) binding. A corollary of this model is that in the presence of Mg·ATP, the inhibitor should be localized at (or close to) the *c* ring/*a* interface. Here we report characterization of PA1b binding to the V-ATPase of the agricultural pest tobacco hornworm (*Manduca sexta*) using structural and biochemical techniques. Using electron microscopy, we show that PA1b binds at the base of the *c* ring, the first direct visualization of inhibitor binding to V-ATPase. In contrast to predictions of existing models, addition of ATP to induce stepping of the V-ATPase rotor failed to localize PA1b into the subunit *a*/*c* ring interface. Instead, biochemical and electron microscopy data indicate that PA1b binds at a site to which both the *e* subunit and *c* ring contribute. This site has some overlap with that for bafilomycin. These results offer new insights into both the structural arrangement of the V-ATPase and characterization of a highly specific inhibitor with pesticidal potential.

## EXPERIMENTAL PROCEDURES

### 

#### 

##### Insect Rearing and Bioassays

*S. oryzae* strains WAA42 and ISOR3 were reared according to Louis *et al.* (25). Toxicity assays with PA1b or bafilomycin were conducted as described previously (15). PA1b labeling using ^125^I and binding assays using the ^125^I toxin were performed according to Ref. 22, and binding data were analyzed using the SIMFIT software. Fifth instar larvae of *M. sexta* (Lepidoptera, Sphingidae), weighing 6–8 g, were reared under long day conditions (16 h of light) at 27 °C using the gypsy moth diet (MP Biomedicals). The *M. sexta* V_1_V_o_ holoenzyme was extracted and purified as described previously (15), which displayed clear and discrete bands on SDS-PAGE (see Fig. 4*A*).

##### PA1b Extraction and Synthesis

Native PA1b was extracted from pea seeds with solvent extraction and HPLC purification. Briefly, pea seeds were ground and the flour dissolved in ethanol 60% (10 ml/g of flour) and incubated under stirring at 4 °C for 2 h. The mixture was then centrifuged at 10,000 × *g* for 10 min and the supernatant dried under vacuum. The resulting powder was resuspended in ethanol (60%) and injected into a reverse phase C_18_ HPLC column (250 × 4.6 mm, 5 μm (Phenomenex), on an Agilent 1200 HPLC) eluted at 1 ml min^−1^. The gradient contained water (with 0.1% TFA)/acetonitrile (with 0.1% TFA) in the ratio 80/20 for 2 min, then 40/60 for 20 min. PA1b peptide isoforms were detected by absorbance at 210 nm, quantified by the measurement of peak area with weighted pure peptide as standards.

The benzophenone moiety was introduced at position 12 to Fmoc-4-benzoyl-l-phenylalanine (Fmoc-l-Bpa), a position shown to not be essential for PA1b binding (26). The variant was synthesized and folded following the optimized procedure described for the production of synthetic PA1b (27), using solid-phase peptide methods and the Fmoc/*t*Bu (*N*-(9-fluorenyl)methoxycarbonyl/*tert*butyl) strategy (24, 25). Purity of the peptide was assessed using RP-HPLC and MALDI-TOF mass spectrometry.

PA1b-biotin was obtained from Proteogenix (Strasbourg, France) and was chemically synthesized with the biotin group attached on the N terminus. PA1b-biotin was folded *in vitro* according to Da Silva *et al.* (27).

##### PA1b Complex Formation

This was conducted using two different protocols. In the first instance, biotinylated PA1b (1 mg ml^−1^) was mixed with streptavidin-HRP ((Thermo Scientific 21126) (5 mg ml^−1^)) and preincubated overnight. A total of 6 μl of this conjugate was mixed with 4 μl of V-ATPase (1 mg of protein ml^−1^) and made up to 60 μl using V-ATPase buffer (150 mm NaCl, 20 mm Tris-HCl, pH 8.1, 9.6 mm 2-mercaptoethanol, 0.01% C_12_E_10_). For the second experiment, 4 μl of V-ATPase (4 μg) was mixed with 3 μl of biotin-PA1b (3 μg) and 3 μl of streptavidin-HRP (15 μg), made up to 60 μl using V-ATPase buffer and incubated for 30 min. Mg·ATP was from a stock solution of 100 mm at pH 7.5 to a final concentration of 5 mm, and the mixture was incubated at room temperature for 5 min to allow for complete turnover.

##### Inhibitor Assays

V-ATPase assays based on the detection of released phosphate using the *M. sexta* V-ATPase or yeast vacuolar membranes were carried out as reported previously (23, 28, 29).

##### Subunit c and e Substitutions

Total RNA was extracted from 80 mg of *S. oryzae* using the RNAspin mini kit (GE Healthcare), and cDNA was obtained with the Moloney murine leukemia virus reverse transcriptase (New England Biolabs). The *S. orizae* V-ATPase subunits *e* and *c* cDNA were obtained by PCR using primers designed according to the sequences found in *S. orizae* sensible strain WAA42 EST (30). The primer pairs were 5′-CTCGAGTTAGTTCAGTGGATTACCCCATGC-3′ (forward)/5′ GGATCCATGGGTGCAGCAGCTTTGCCTTTTAT-3′ (reverse) and 5′-AACATGGGGGTGGGAATTGT-3′ (forward)/5′-TCAGTGCTGTGTTGTGCACCT-3′ (reverse), respectively. The *e* and *c* cDNA were inserted in the yeast expression vector pYES2 (Invitrogen) between the XhoI and BamHI restriction sites. Plasmids carrying the coding sequence for *S. orizae* V-ATPase subunits *e* or *c* were used to transform by electroporation *Saccharomyces cerevisiae* strains based on strain BY474 (Mat a, *his3*Δ1, *leu2*Δ0; *met15*Δ0, *ura3*Δ0) that were, respectively, deleted for YCL005w-A (*VMA9*::kanMX4) or YEL027w (*VMA3*::kanMX4), obtained from Euroscarf (Frankfurt, Germany). For expression of the heterologous gene, yeast were grown on selective galactose medium (0.7% w/v YNB, 2% w/v galactose, 2% w/v agar, 0.002% w/v histidine, 0.006% w/v leucine, and 0.006% w/v methionine). The analysis of pH dependence of growth was carry as described in (31). PA1b was added onto the medium to test the effect of toxicity at 1 mg ml^−1^.

##### Photoaffinity Labeling

For labeling, *M. sexta* V_1_V_o_ holoenzyme (30 μg), V_1_ complex (20 μg), or V_o_ complex (10 μg) in 80 μl of V-ATPase buffer were mixed with 1 μl ^125^I-PA1b-benzophenone (10 kilobecquerels μl^−1^ determined by scintillation counting) and incubated for 5 min at 20 °C. Cross-linking was induced by irradiating the samples for 10 min with UV light on ice. Subsequently, samples were separated by SDS-PAGE and stained with Coomassie. The gels were then dried on Whatman paper, exposed to a phosphorimaging screen, and analyzed with a PhosphorImager (GE Healthcare). Afterward the lanes of the dried gel were cut into approximately equally sized slices, including regions where no Coomassie-stained proteins were visible. Each piece was mixed with 5 ml of scintillation liquid, and the disintegrations were counted (Beckman LS6500 scintillation counter) up to 10,000 counts for each sample to ensure a S.D. of 1%.

##### Electron Microscopy

Negative stain grids were prepared by applying 3 μl of protein solution (∼40 μg ml^−1^) onto a carbon-coated copper grid that had previously been placed for 40 min under a UV lamp (32). The grid was then stained with 1% w/v uranyl acetate. Grids were imaged using a Technai T12 microscope fitted with a LaB6 source operating at 120 kV. Micrographs were recorded on a 2000 ×2000 Gatan CCD camera. Particles were hand-picked using the Boxer program in EMAN2 (33). Particles were centered, masked, and classified in IMAGIC-5, resulting in a crude set of reference images, which were used for multireference alignment (34). The classes produced were significantly improved, and representative ones were used for a further stage of multireference alignment. This process was iterated until no further improvement was observed. Poorly aligning particles were removed.

Three-dimensional reconstructions were generated in EMAN using the previously solved intact *M. sexta* V-ATPase reconstruction filtered to 50 Å as an initial starting model. The resulting three-dimensional reconstruction showed clear differences to the starting model at the base of V_o_. An additional model of the negatively stained *M. sexta* V-ATPase was also generated as a control to ensure that the features seen were not artifacts of staining. Because only ∼20% of the V-ATPase particles showed binding, models were generated using in the first instance those particles which showed a clear PA1b/streptavidin-HRP density, along with representative particles of the V-ATPase in orientations for which the PA1b was not visible. In a second experiment, these models were used for multimodel refinement using both an apparent PA1b-bound model and an unbound model, both filtered to 50 Å. Unsupervised multimodel refinement was carried out on the full data sets in both EMAN multimodel mode and Xmipp ML3D classification mode to check that selection of particles was not biasing the resulting models (35, 36). The resulting independently generated reconstructions were of equivalent quality and showed features consistent with PA1b binding.

## RESULTS

### 

#### 

##### Benzophenone and Biotin Derivatives of PA1b Remain Effective V-ATPase Inhibitors

**FIGURE 2.**
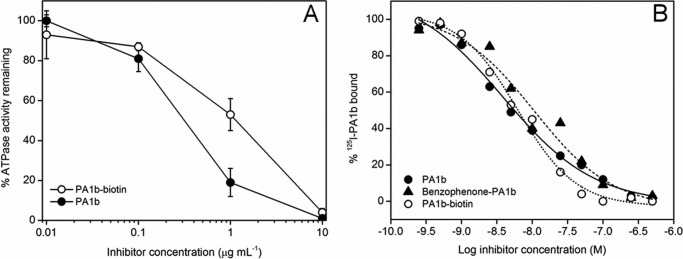
**Activities of modified PA1b.** *A*, inhibition of the ATPase activity of *M. sexta* V-ATPase by PA1b and PA1b-biotin. *B*, competitive binding of ^125^I-PA1b (0.4 nm) at increasing concentrations of PA1b (*filled circles*), chemically synthesized PA1b-biotin (*open circles*), or benzophenone-PA1b (*filled triangles*). Binding was to a crude membrane extract of *S. oryzae*. The nonspecific binding was determined by adding a large amount of purified PA1b (1 μm) and was 25–35% of total binding.

Two PA1b derivatives were used for this study. These were substituted either by a benzophenone photoactivable group or by a biotin group. Measurement of *M. sexta* V-ATPase activity in the presence of the PA1b-biotin derivative showed no significant difference in activity from native PA1b (Fig. 2*A*). Competitive inhibition of ^125^I-PA1b binding to a *S. oryzae* membrane extract by the modified PA1b revealed no significant difference in the binding of PA1b, PA1b-benzophenone or PA1b-biotin (*K_i_* of 6.5 ± 1.13 nm, 11 ± 2.2 nm, and 5.4 ± 6.6 nm, respectively (Fig. 2*B*)).

##### Identifying the PA1b Binding Site by Electron Microscopy

**FIGURE 3.**
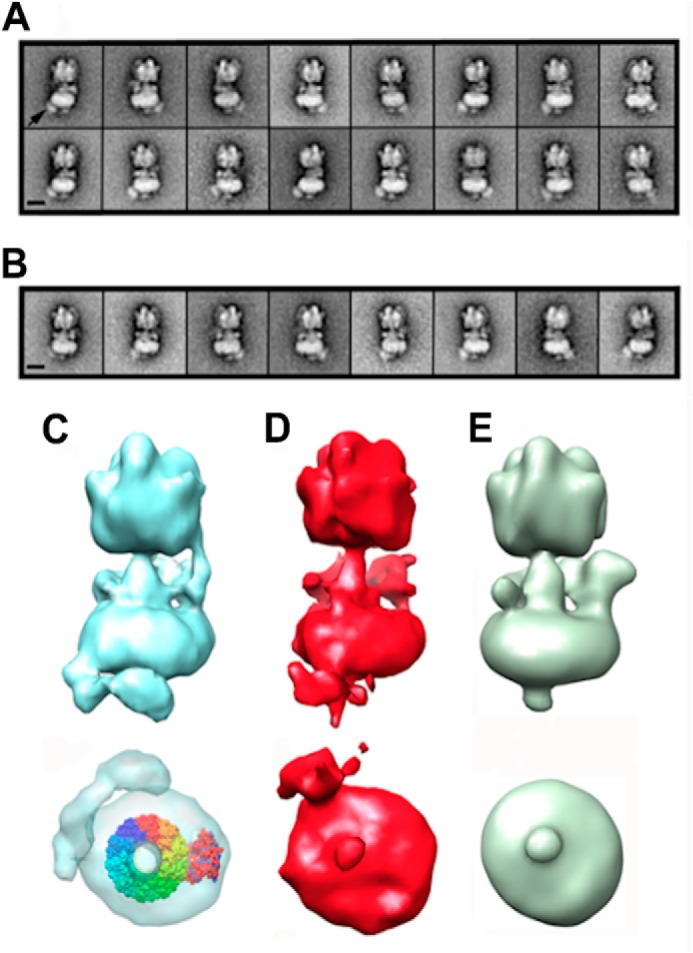
**Electron microscopy of PA1b-bound V-ATPase.** *A*, representative classes of PA1b-streptavidin-HRP-bound V-ATPase in the absence of ATP. *B*, as *A* but in the presence of 2 mm Mg·ATP. The PA1b-streptavidin-HRP density is indicated by an *arrow* in the *far left panel 1* of *A. Scale bars* in both *A* and *B* represent 15 nm. *C–E*, three-dimensional reconstructions of the V-ATPase viewed perpendicular to the long axis of the complex (*upper image*) and from the extracellular end (*lower image*) bound to PA1b (*C*), bound to PA1b after the addition of Mg·ATP (*D*) and a control with no PA1b (*E*). All models were generated using EMAN, and the picture was produced using Chimera rendered at the same sigma level. In *C* (*lower*), the decameric c ring (Protein Data Bank ID code 2DB4 (53) r*ainbow colors*) and *a* subunit model (*red*) have been fitted to the PA1b-streptavidin-HRP V-ATPase reconstruction in the absence of ATP using Chimera. If catalytically active, the *c* ring would rotate counterclockwise with respect to subunit *a* when observed from this perspective.

Negative stain grids showed the V-ATPase to be monodisperse, with little dissociation of the complex. Image class averages were well defined and showed clear stator connections. To reduce any bias in data processing, in the first instance six samples were prepared: 1, V-ATPase; 2, V-ATPase with streptavidin-HRP; 3, V-ATPase with 5% ethanol (control for the PA1b solvent); 4, V-ATPase with biotin-PA1b; 5, V-ATPase with simultaneously added biotin-PA1b and streptavidin-HRP and 6, V-ATPase with biotin-PA1b/streptavidin-HRP complex preformed by overnight conjugation. The electron microscopy data were then collected at ×23,000 magnification and processed blind to minimize bias. In total, 8644, 8543, 6538, 8006, 9712, and 5740 particles were picked using EMAN2 for samples 1, 2, 3, 4, 5, and 6, respectively. The resulting classes showed that only two samples displayed extra density about the base of V_o_ (supplemental Fig. 1). These samples were the only ones to contain the full PA1b streptavidin-HRP complex, with no notable differences being detected with preformed complex or simultaneous addition of the label components (samples 5 and 6). Analysis of the data showed that ∼20% of V-ATPase particles were labeled. To improve resolution, data were collected at ×30,000 magnification, giving 10,056 particles from 527 micrographs. Additional grids were prepared for V-ATPase with Mg·ATP with data also collected at ×30,000 magnification, with 841 micrographs providing 11,341 particles. Particles that aligned poorly, that were unstable during classification or produced obviously degraded or nonrepresentative views were removed, resulting in 6720 and 7355 particles for the inactive and ATP-treated specimens, respectively. Both datasets gave distinctive classes in which PA1b binding could clearly be identified at the base of V_o_ (Fig. 3, *A* and *B*).

To further characterize the PA1b binding site, three-dimensional reconstructions were generated for PA1b-labeled V-ATPase ± Mg·ATP and compared with a control without inhibitor (Fig. 3, *C–E*). In the absence of ATP, the resulting reconstruction showed extra density distributed in a ∼115° arc around the base of V_o_ (Fig. 3*C*), starting near stator filaments 2 and 3 which flank subunit *c* (11). The most plausible interpretation of this labeling pattern is that binding occurs toward the extracellular surface of the *c* ring. Based on the organization of the homologous decameric NtpK ring (37), this could be in the region of the C-terminal ends of transmembrane helices 2 or 4 of the subunit *c* four-helical bundle, or in the extracellular loop linking helices 2 and 3. The distribution of label suggests that labeling occurred at any one of 3–4 equivalent (and adjacent) sites on the *c* ring. Strikingly, after addition of Mg·ATP, the density was restricted to an arc of only ∼30° (Fig. 3*D*), suggesting that binding was limited to only a single subunit *c* site. Although the reconstructions are of modest resolution and quality, it is clear from both the classes and reconstructions that in both the absence and presence of ATP, PA1b binding is distant from the *a* subunit/*c* ring interface, the proposed site of inhibitor action for other molecules targeting the V-ATPase membrane domain (14, 24). Models produced by EMAN and Xmipp were consistent, both in single-model refinement mode and after unsupervised multimodel refinement.

##### PA1b Binding to the e and c Subunits

**FIGURE 4.**
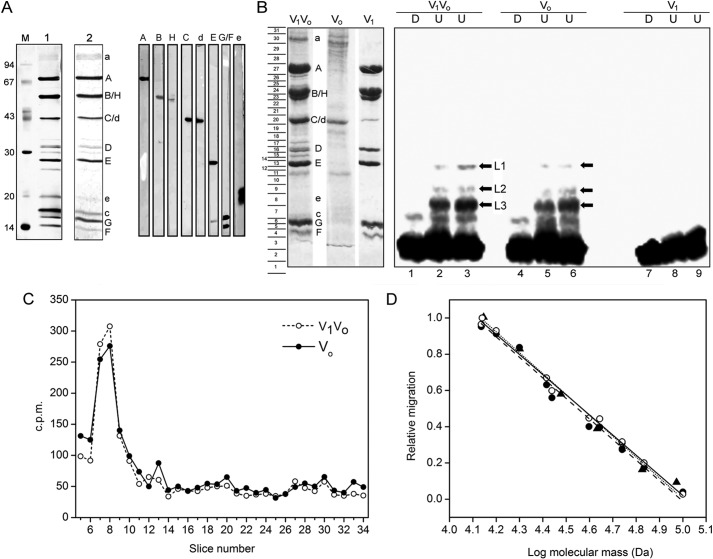
**Characterization of PA1b binding.** *A*, *left*, SDS-PAGE analysis of purified *M. sexta* V-ATPase with staining with silver (*lane 1*) or Coomassie Brilliant Blue (*lane 2*) and *M* indicating molecular mass markers. *Right*, identification of the staining polypeptides by immunoblot analysis. *B*, photoaffinity labeling of *M. sexta* V-ATPase with the ^125^I-PA1b-benzophenone. For labeling, V_1_V_o_ holoenzyme (V_1_V_o_), V_o_ complex (V_o_), or V_1_ complex (V_1_) was incubated with ^125^I-PA1b-benzophenone and exposed to UV light or kept in the dark. After separation by SDS-PAGE, the stained and dried gel was exposed to a phosphorimaging screen. *Left*, SDS-PAGE of the V-ATPase complexes with Coomassie Blue staining. Right, readout of the phosphorimaging screen for labeled holoenzyme (*lanes 1–3*), V_o_ (*lanes 4–6*), and V_1_ (*lanes 7–9*) after either UV irradiation (*U*) or maintenance in the dark (*D*). Bands L1–L3 (*arrows*) indicate UV-dependent labeled species. *C*, slices of the dried gel from samples of the V_1_V_O_ holoenzyme (*open circles*) and V_o_ (*filled circles*) subjected to scintillation counting. The column to the *left* of *B* indicates positions of gel slices subjected to counting. The majority of the radioactivity was found in *slice 3* near the dye front. *D*, molecular mass determination of ^125^I-labeled V-ATPase subunits. A calibration curve was prepared by plotting log molecular masses of standard proteins (*triangles*, *dashed line*) and V-ATPase subunits (deduced from their primary structure) against their relative migration on the SDS-polyacrylamide gels shown in *A* (*open circles*, *dotted line*) and *B* (*filled circles*, *solid line*). In each case, linear regression gave *r*^2^>0.99.

Independent confirmation of the binding of PA1b to subunits at the base of V_o_ was provided by photoaffinity labeling with the ^125^I-labeled benzophenone derivative of PA1b. After UV irradiation, covalent attachment of the modified inhibitor to purified V_1_V_o_ holoenzyme or the separated V_o_ or V_1_ domains from *M. sexta* was detected after SDS-PAGE by autoradiography of the dried gel. In the gel system used, all subunits of the holoenzyme were resolved (Fig. 4*A*, *left*), and their positions were identified by immunoblotting (Fig. 4*A*, *right*). Note that subunits *d* and *e* stain only weakly with Coomassie Blue (Fig. 4*A*, *lane 2*) but can be located after staining with silver (Fig. 4*A*, *lane 1*). The autoradiography showed UV irradiation-dependent labeling of three species (identified as L1–L3 in Fig. 4*B*, *right*) in both the holoenzyme and in V_o_, but not in the soluble catalytic domain V_1_. To confirm localization of radioactivity to these regions of the gel, the dried gel was cut into slices as indicated in Fig. 4*B* and disintegrations of the slices determined by scintillation counting (Fig. 4*C*). The applied 10 kilobecquerels was almost completely recovered, and as expected, the majority of the radioactivity was in slice 3 (data not shown) which is just above the dye front after electrophoresis. The next most radioactive slices were numbers 7 and 8, reflecting the approximate position of the V_o_ subunit *e*, as indicated by silver staining and immunoblotting of the comparable nonradioactive sample (Fig. 4*A*).

To determine the masses of the ^125^I-PA1b-labeled species, a calibration curve of molecular mass *versus* relative migration on SDS-PAGE was constructed (Fig. 4*D*). Masses of V-ATPase subunits were determined from their primary structures (with the exception of the *e* subunit that has an actual mass of 9.7 kDa but apparent mass of 20 kDa because of extensive glycosylation (49)). Masses of radiolabeled species L1, L2, and L3 were determined to be 27.5, 21.6, and 18.4 kDa, respectively. Given that labeling occurs exclusively at the V_o_ membrane domain containing only *a*, *c*, *d*, and *e* subunits, the radioactively labeled band L3 can be identified as subunit *c* (mass of 15.8 kDa) with a PA1b adduct adding an apparent additional mass of 2.5 kDa. Similarly, band L2 is most likely to be subunit *e* (mass ≈20 kDa with a 1.6-kDa PA1b adduct. Band L1 can be assigned as a dimer of subunit *c*; although the deduced mass of 27.5 kDa (including a PA1b adduct) is significantly less than double the mass of subunit *c*, dimers of this type of polypeptide have been observed to migrate anomalously quickly during SDS-PAGE (38). The ratio of intensity of bands L2 and L3 reflects the likely 10:1 *c*:*e* stoichiometry.

##### Expression of Insect c or e Subunit in Saccharomyces Is Not Sufficient to Confer Sensitivity to PA1b

**FIGURE 5.**
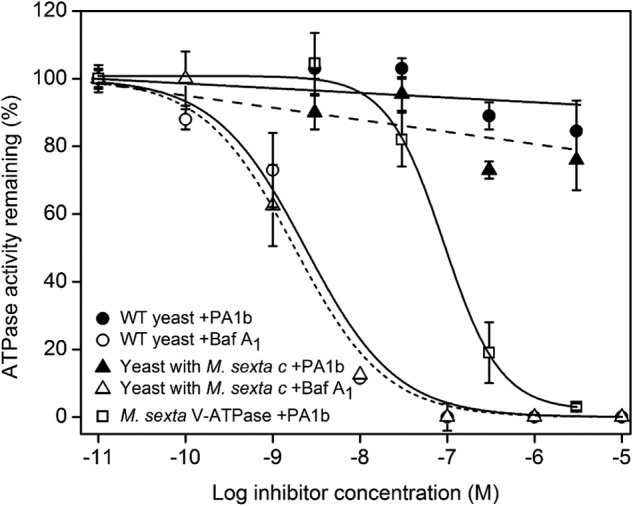
**Inhibition of yeast V-ATPase activity.** V-ATPase activity from the wild type *S. cerevisiae* strain BMA64-1B (WT) and BMA64-1B deleted for the *VMA3* gene but expressing the *M. sexta c* subunit was measured in the presence of bafilomycin A_1_ (*open circles*) and PA1b (*filled circles*). For comparison, inhibition of the purified V_1_V_o_ holoenzyme of *M. sexta* (*squares*) by PA1b is included.

If insect *c* and *e* subunits constitute the binding site for PA1b, it could be predicted that heterologous expression of these polypeptides in a suitable host might confer sensitivity of the host V-ATPase to PA1b. Expression of *M. sexta* subunit *c* in a *S. cerevisiae* strain deleted for the *VMA3* gene encoding *M. sexta* subunit *c* (14) did lead to complementation of the pH-sensitive conditional lethal phenotype characteristic of *vma* mutants (39) indicated by growth of transformed cells at pH 7.5, and hence the insect *c* subunit must be incorporated into a functional V-ATPase. However, although the *M. sexta* V-ATPase is inhibited by PA1b with an apparent *K_i_* of ∼91 nm (Fig. 5), expression of the insect *c* subunit did not confer PA1b sensitivity to yeast (Fig. 5). Wild type and *M. sexta c*-expressing yeast strains both produced V-ATPase activity that was equally sensitive to bafilomycin (*K_i_* ∼2.4 nm).

Similarly, although the *S. oryzae c* subunit complemented the *vma3* mutation, resulting in wild type levels of growth at pH 7.5, transformed cells were not sensitive to PA1b (data not shown). Growth of the *e* subunit mutant on pH 7.5 medium showed no difference between the mutant and wild type. However, experiments using ^125^I-PA1b showed no binding activity on membrane extracts from transformed yeast expressing either *c* or *e* subunit from *S. oryzae*.

##### PA1b-resistant S. oryzae Are Partially Resistant to Bafilomycin

**FIGURE 6.**
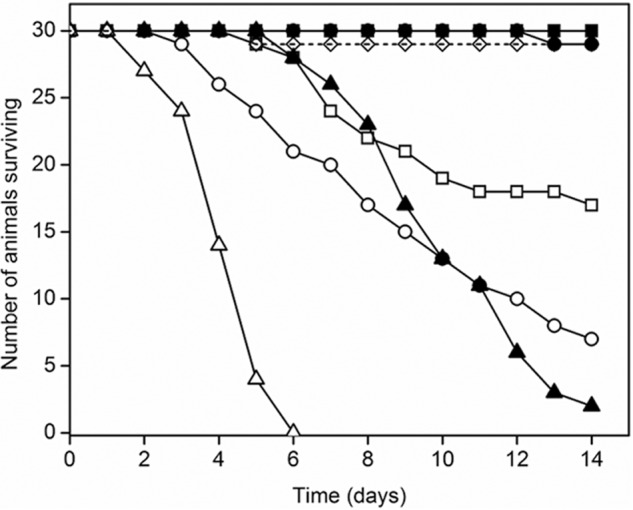
**Toxicity of the V-ATPase inhibitor bafilomycin toward PA1b-resistant *S. oryzae*.** 30 rice weevils (*S. oryzae*) were fed on artificial diet incorporating each tested compound over 14 days. The weevil strains were WAA42 (PA1b-sensitive strain: *open symbols*) and ISOR3 (PA1b-resistant strain: *filled symbols*). Bafilomycin was included in food at 0.36 (*squares*), 0.60 (*circles*), or 2.00 mg/g food and survival over a 14-day period monitored. *Open diamonds*/*dashed line*, control for artificial diet with bafilomycin solvent.

Assays of *in vivo* sensitivity to bafilomycin of two cereal weevil strains were performed by adding the inhibitor to the food supply. Animals of PA1b-sensitive strain WAA42 were also sensitive to bafilomycin at 0.36 and 0.60 mg/g of food, with deduced TL_50_ of 12.6 ± 2.3 and 9.4 ± 1 days, respectively (Fig. 6). In contrast, no mortality was observed with the PA1b-resistant strain ISOR3 at these doses. Only at a dose of 2.0 mg/g food were the PA1b-resistant weevils killed by bafilomycin, but with a TL_50_ of 9.9 ± 0.5 days for ISOR3 animals compared with 4.0 ± 0.2 days for WAA42. Moreover, even up to 10 μm bafilomycin was unable to inhibit binding of radiolabeled PA1b to WAA42 membrane extracts (data not shown).

## DISCUSSION

The V-ATPase is an important therapeutic target principally because of its roles in cancer cell survival and bone resorption (40, 41). Because tumor cell V-ATPase is essential for regulation of cytoplasmic pH and acidification of the extracellular environment, V-ATPase inhibitors induce apoptosis and loss of invasive phenotype (41). Inhibition of activity in osteoclasts decreases bone resorption, hence inhibitors have also been assessed at length as anti-osteoporotic drugs (13). The central role of the V-ATPase in insect gut physiology (42) means that the enzyme is also valuable as a potentially novel pesticide (43). There is also potential for development of anti-malarials targeting the *Plasmodium* parasite (44, 45) or its mosquito vector (46). Development of lead compounds should be achievable through targeting isoforms highly expressed in particular cells or tissues, such as the a_3_ isoform in osteoclasts. However, although some selectivity has been reported (47), the ubiquitous nature of the V-ATPase has limited identification of highly selective inhibitors. The peptidic inhibitor PA1b is therefore remarkable, not only because of its V-ATPase specificity and potency, but also because it is uniquely selective against the enzyme from insects (17, 19). To date, the precise interaction of PA1b with the V-ATPase has remained unexplained. The data presented here provide a much clearer picture of binding and hence the mechanism of inhibition, opening the door to further development.

Previous work has shown that PA1b binds to V_o_ which consists only of subunits *a*, *d*, *e* and the *c* ring (23). Although this implies an effect on proton translocation, assignment of binding to a particular subunit could not be made. Consequently, conclusions about the mode of PA1b action were also limited. In this work, novel use of a streptavidin-HRP tag has allowed visualization of PA1b binding to the V-ATPase by electron microscopy. PA1b at 3.7 kDa is too small to visualize directly at the level of resolution attainable by negative stain electron microscopy. Visualization was instead made by attaching a streptavidin-HRP tag to biotinylated PA1b that is readily detected in the electron microscope. Crucially, the biotin linker is sufficiently short and the tag structure sufficiently compact that the streptavidin-HRP density provides an accurate indicator of the site of PA1b binding.

Classes and reconstructions both clearly show PA1b binding to the base of V_o_, a surface that is extracellular to the plasma membrane of the *Manduca* midgut. Only four polypeptides are recognized components of V_o_ (with likely stoichiometry *a*:*c*_10_:*d*:*e*). The data presented here exclude binding to *d*, the ∼40-kDa subunit that couples the axle of the V-ATPase rotor to the *c* ring on the cytoplasmic side of the membrane (48). The data also appear to exclude binding to subunit *a*, the membrane subunit that forms the asymmetric mass within V_o_. Binding to the *c* ring or to *e* can therefore be inferred, and contact with both is in fact supported by photochemical labeling data. These show V_o_ components of masses approximating only to those of *c* and *e* shifted on SDS-PAGE, by the attached inhibitor. The apparent masses of PA1b adducts required to be added to subunits *c* and *e* to give the radiolabeled bands 2 and 3 in Fig. 4 are neither equal in both cases nor equivalent to the actual mass of PA1b (3.7 kDa). However, this is not unexpected because a shift in migration on SDS-polyacrylamide gel rarely reflects the simple sum of the masses of adduct and target polypeptide. Instead, migration will likely be influenced by both the position of the cross-linking adduct and its impact on the chemical character of the target polypeptide. In this respect, it is worth noting that both *c* and *e* migrate anomalously because they are highly hydrophobic and extensively glycosylated, respectively.

Based on the crystal structure of the homologous NtpK ring, *M. sexta* subunit *c* is modeled as a four-transmembrane helix bundle, with both its N and C termini on the luminal/extracellular side of the membrane (29). Assembled as a ring of 6–10 subunits, helices 1 and 3 alternate to form an inner ring with helices 2 and 4 forming an outer ring in contact with the lipid phase. Based on this model and the available electron microscopy data, the regions of subunit *c* to which PA1b most likely binds are the extreme N and C termini and the extracellular loop linking helices 2 and 3. These regions do not appear to make contact with other subunits, hence their sequences are least conserved in the otherwise generally highly conserved polypeptide. This variability may explain the remarkable selectivity of PA1b for the insect protein.

The position of subunit *e* in V_o_ is much less certain. This 88-residue polypeptide is predicted to fold with two transmembrane helices and carries extensive *N*-linked glycosylation (49). A consensus glycosylation site is present toward its C terminus (Asn-68), implying that both termini could be on the luminal/extracellular side of the membrane. Presumed to be part of the stator in association with subunit *a*, its assembly into the rotor of the complex has not been excluded. The data presented here indicate that subunits *e* and *c* must be in close proximity because both are labeled by PA1b, and two separate binding sites on completely different polypeptides seem unlikely. The *e*/*c* contact could be either static, with both subunits forming parts of the rotor, or via a stator-rotor interface that is dynamic during catalysis. To further clarify binding, we introduced PA1b-sensitive insect *c* and *e* subunit into yeast strains disrupted for the corresponding homologues. Parent strains of these yeasts are insensitive to PA1b (23). In both cases, although the insect proteins complemented the pH-sensitive conditionally lethal phenotype of the deletion strains (restoring bafilomycin-sensitive activity), there was no detectable increase in sensitivity to PA1b. These data indicate that neither the *e* or *c* subunit alone is sufficient to form the PA1b binding site, suggesting that both subunits are required. Unfortunately, a *VMA3*/*VMA9* double knock-out yeast strain could not be produced to test this further.

Lipophilic molecules such as bafilomycin, archazolid, and apicularen bind to the lipid-exposed surface of subunit *c* (14, 15, 29). The additional role of subunit *a* in the inhibitory effects of bafilomycin and apicularen (14, 24) implies that the inhibitor links *c* to *a* at the rotor-stator interface, obstructing stepping of the rotor and preventing proton translocation. A prediction of this model is that for a *c* ring carrying a single inhibitor molecule, rotation energized by the catalytic cycle of the enzyme should propel the inhibitor-loaded *c* subunit to the stator interface, allowing it to contact subunit *a*. Thus, in the presence of ATP, inhibitor should localize to a point adjacent to the stator. Using the image averaging method applied here, this would equate to PA1b no longer appearing to be distributed about multiple equivalent positions, with the mass instead becoming focused at a single location. This prediction was partly substantiated, with bound PA1b apparently becoming restricted to a single *c* subunit when it was present during catalytic activity. However, its location distant from the asymmetric density that represents subunit *a* in the classes was surprising, clearly inconsistent with the models of inhibitor action outlined above. This leads to a number of possibilities. First, the inhibition model is correct, but the membrane-bound part of the V-ATPase stator extends significantly further around the *c* ring than previously suspected, such that the inhibitor label seen in Fig. 4*D* is actually bound to the *c* ring/stator interface. Subunit *e* could be part of this interface, explaining its photochemical labeling. However, based on the expected direction of *c* ring rotation (counterclockwise in Fig. 4, *C* and *D*), the inhibitor label in Fig. 4*D* would be closest to the point at which the rotating *c* ring exits the interface with subunit *a*, not enters it. To accommodate this, PA1b would have to prevent the *c* ring from stepping out of its interface with the stator rather than obstructing its entry. A second possibility is that PA1b binds only to *c* subunits that have a particular conformation affected by the catalytic cycle of the enzyme. This is speculative, but conformational variability could also explain why PA1b binds only a discrete number of adjacent *c* subunits to one side of the *c* ring. It is clear from electron microscopy that not all potential sites on the *c* ring are occupied by PA1b even at high inhibitor/enzyme ratios. This suggests that characteristics of individual *c* ring subunits could vary according to their position with respect to subunit *a*.

An unexpected result was obtained when the sensitivity to bafilomycin of PA1b-resistant cereal weevil strains was examined. There is clear correlation between resistance *in vivo* to PA1b and bafilomycin, implying significant overlap in their binding sites. However, PA1b binding was not inhibited by bafilomycin even at concentrations ∼10,000-fold higher than its IC_50_. Our electron microscopy data indicate that PA1b exerts its effect away from subunit *a*, whereas bafilomycin is presumed to act at the *a*/*c* subunit interface. Therefore we presume that the common element for bafilomycin and PA1b toxicity is the *c* subunit. It has been shown previously that PA1b binds with high affinity to extracts from susceptible *S. oryzae* but that there is no detectable binding to equivalent extracts from resistant weevils (22). Consequently, resistance is due to modification of the PA1b-binding protein rather than for example, enhanced catabolism of inhibitor. Sequences of *S. oryzae* subunit *c* show only two amino acid changes in four PA1b-resistant strains that have been examined: Ser-83 in the extracellular loop between helices 2 and 3 is replaced by Asn, and Ala-159 at the extracellular C-terminal end of helix 4 is replaced by Thr. In models of subunit *c* these residues are in close proximity and in the region identified as PA1b binding by the electron microscopy data. Notably, *e* subunits are identical in susceptible and resistant animals.^5^ Bafilomycin and PA1b may have a close but different binding site on the *c* subunit, such that bafilomycin does not directly compete with ^125^I-PA1b. However, the sequence change between the PA1b-sensitive and -resistant weevils can be sufficient to exert a relatively long range effect on the bafilomycin binding site. In this regard, mutations to fungal *c* subunits that correlate with bafilomycin resistance include changes to residues that are some distance from the proposed binding site or at locations within the packed helical bundle (50). Hence, relatively long range or indirect effects on helical packing can impact on bafilomycin binding. Investigations are underway to understand increased resistance.

PA1b has activity against a number of pests, including cereal weevils and mosquitoes, but to date it appears to be exclusively insecticidal (17, 19). Sensitivity of insects to loss of V-ATPase activity, using for example an oral RNAi approach (43, 51), underline the potential of the complex as a potential target for insecticides. Characterization of PA1b binding to insect V-ATPase and explanation of its action will facilitate study of its insecticidal potential. Inhibitors acting on the membrane domains of the V- and F-ATPases may be unique in acting to obstruct the rotary motion on which these enzymes depend. Macrolide antibiotics such as bafilomycin are relatively lipid-soluble (52); hence, they access V_o_ from the lipid phase and can permeate into all membrane compartments in the cell. Binding of PA1b to the surface of the V-ATPase rotor exposed on the external surface of the cell gives it a further unique property, the ability to inhibit without entering the cell. This property explains how PA1b targets plasma membrane forms of the V-ATPase and why the peptide is such a potent and effective oral inhibitor.

PA1b has potential to be the leading compound of a new insecticide class. Optimization of the interactions it makes with V-ATPase could help increase toxicity or even provide a template from which to develop new insecticides. Thus, our study has not only revealed important aspects of V-ATPase structure and function, but can also inform development of novel inhibitors to treat a range of diseases.

## Supplementary Material

Supplemental Data

## Footnotes

5 D. Pauron and F. Gressent, unpublished data.

## Footnotes

4 The abbreviations used are:

V-ATPase: vacuolar H^+^-ATPase
Fmoc: *N*-(9-fluorenyl)methoxycarbonyl
PA1b: pea albumin 1 subunit b.

## References

[1] Forgac M. (2007) Vacuolar ATPases: rotary proton pumps in physiology and pathophysiology. Nat. Rev. Mol. Cell Biol. 8, 917–929 17912264 10.1038/nrm2272

[2] Beyenbach K. W. Wieczorek H. (2006) The V-type H^+^ ATPase: molecular structure and function, physiological roles and regulation. J. Exp. Biol. 209, 577–589 16449553 10.1242/jeb.02014

[3] Karet F. E. Finberg K. E. Nelson R. D. Nayir A. Mocan H. Sanjad S. A. Rodriguez-Soriano J. Santos F. Cremers C. W. Di Pietro A. Hoffbrand B. I. Winiarski J. Bakkaloglu A. Ozen S. Dusunsel R. Goodyer P. Hulton S. A. Wu D. K. Skvorak A. B. Morton C. C. Cunningham M. J. Jha V. Lifton R. P. (1999) Mutations in the gene encoding B1 subunit of H^+^-ATPase cause renal tubular acidosis with sensorineural deafness. Nat. Genet. 21, 84–90 9916796 10.1038/5022

[4] Capparelli R. Palumbo D. Iannaccone M. Iannelli D. (2009) Human V-ATPase gene can protect or predispose the host to pulmonary tuberculosis. Genes Immun. 10, 641–646 19536151 10.1038/gene.2009.48

[5] Li Y. P. Chen W. Liang Y. Li E. Stashenko P. (1999) Atp6i-deficient mice exhibit severe osteopetrosis due to loss of osteoclast-mediated extracellular acidification. Nat. Genet. 23, 447–451 10581033 10.1038/70563

[6] Sennoune S. R. Martinez-Zaguilan R. (2007) Plasmalemmal vacuolar H^+^-ATPases in angiogenesis, diabetes and cancer. J. Bioenerg. Biomembr. 39, 427–433 18058006 10.1007/s10863-007-9108-8

[7] Imamura H. Nakano M. Noji H. Muneyuki E. Ohkuma S. Yoshida M. Yokoyama K. (2003) Evidence for rotation of V_1_-ATPase. Proc. Natl. Acad. Sci. U.S.A. 100, 2312–2315 12598655 10.1073/pnas.0436796100 PMC151337

[8] Yokoyama K. Nakano M. Imamura H. Yoshida M. Tamakoshi M. (2003) Rotation of the proteolipid ring in the V-ATPase. J. Biol. Chem. 278, 24255–24258 12707282 10.1074/jbc.M303104200

[9] Muench S. P. Trinick J. Harrison M. A. (2011) Structural divergence of the rotary ATPases. Q. Rev. Biophys. 44, 311–356 21426606 10.1017/S0033583510000338

[10] Kitagawa N. Mazon H. Heck A. J. Wilkens S. (2008) Stoichiometry of the peripheral stalk subunits E and G of yeast V_1_-ATPase determined by mass spectrometry. J. Biol. Chem. 283, 3329–3337 18055462 10.1074/jbc.M707924200

[11] Muench S. P. Huss M. Song C. F. Phillips C. Wieczorek H. Trinick J. Harrison M. A. (2009) Cryo-electron microscopy of the vacuolar ATPase motor reveals its mechanical and regulatory complexity. J. Mol. Biol. 386, 389–399 10.1016/j.jmb.2009.01.014 19244615

[12] Supino R. Petrangolini G. Pratesi G. Tortoreto M. Favini E. Bo L. D. Casalini P. Radaelli E. Croce A. C. Bottiroli G. Misiano P. Farina C. Zunino F. (2008) Antimetastatic effect of a small-molecule vacuolar H^+^-ATPase inhibitor in *in vitro* and *in vivo* preclinical studies. J. Pharmacol. Exp. Ther. 324, 15–22 17909082 10.1124/jpet.107.128587

[13] Farina C. Gagliardi S. (1999) Selective inhibitors of the osteoclast vacuolar proton ATPase as novel bone antiresorptive agents. Drug Discovery Today 4, 163–172 10322275 10.1016/S1359-6446(99)01321-5

[14] Osteresch C. Bender T. Grond S. von Zezschwitz P. Kunze B. Jansen R. Huss M. Wieczorek H. (2012) The binding site of the V-ATPase inhibitor apicularen is in the vicinity of those for bafilomycin and archazolid. J. Biol. Chem. 287, 31866–31876 22815478 10.1074/jbc.M112.372169 PMC3442520

[15] Huss M. Ingenhorst G. König S. Gassel M. Dröse S. Zeeck A. Altendorf K. Wieczorek H. (2002) Concanamycin A, the specific inhibitor of V-ATPases, binds to the V_o_ subunit *c*. J. Biol. Chem. 277, 40544–40548 12186879 10.1074/jbc.M207345200

[16] Huss M. Wieczorek H. (2009) Inhibitors of V-ATPases: old and new players. J. Exp. Biol. 212, 341–346 19151208 10.1242/jeb.024067

[17] Gressent F. Duport G. Rahioui I. Pauchet Y. Bolland P. Specty O. Rahbe Y. (2007) Biological activity and binding site characteristics of the PA1b entomotoxin on insects from different orders. J. Insect Sci. 7, 1–10 20331395 10.1673/031.007.1201 PMC2999418

[18] Delobel B. Grenier A. M. Gueguen J. Ferrasson E. Mbaiguinam M. (5 11, 1998) French Patent 98 05877

[19] Gressent F. Da Silva P. Eyraud V. Karaki L. Royer C. (2011) Pea albumin 1 subunit b (PA1b), a promising bioinsecticide of plant origin. Toxins 3, 1502–1517 22295174 10.3390/toxins3121502 PMC3268454

[20] Jouvensal L. Quillien L. Ferrasson E. Rahbé Y. Guéguen J. Vovelle F. (2003) PA1b, an insecticidal protein extracted from pea seeds (*Pisum sativum*): ^1^H-2-D NMR study and molecular modeling. Biochemistry 42, 11915–11923 14556622 10.1021/bi034803l

[21] Grenier A. M. Mbaiguinam M. Delobel B. (1997) Genetical analysis of the ability of the rice weevil *Sitophilus oryzae* (Coleoptera, Curculionidae) to breed on split peas. Heredity 79, 15–23

[22] Gressent F. Rahioui I. Rahbé Y. (2003) Characterization of a high affinity binding site for the pea albumin 1b entomotoxin in the weevil *Sitophilus*. Eur. J. Biochem. 270, 2429–2435 12755698 10.1046/j.1432-1033.2003.03611.x

[23] Chouabe C. Eyraud V. Da Silva P. Rahioui I. Royer C. Soulage C. Bonvallet R. Huss M. Gressent F. (2011) New mode of action for a knottin protein bioinsecticide pea albumin 1 subunit b (PA1b) is the first peptidic inhibitor of V-ATPase. J. Biol. Chem. 286, 36291–36296 21890633 10.1074/jbc.M111.281055 PMC3196078

[24] Wang Y. Inoue T. Forgac M. (2005) Subunit a of the yeast V-ATPase participates in binding of bafilomycin. J. Biol. Chem. 280, 40481–40488 16216877 10.1074/jbc.M509106200

[25] Louis S. Delobel B. Gressent F. Duport G. Diol O. Rahioui I. Charles H. Rahbé Y. (2007) Broad screening of the legume family for variability in seed insecticidal activities and for the occurrence of the A1b-like knottin peptide entomotoxins. Phytochemistry 68, 521–535 17222873 10.1016/j.phytochem.2006.11.032

[26] Da Silva P. Rahioui I. Laugier C. Jouvensal L. Meudal H. Chouabe C. Delmas A. F. Gressent F. (2010) Molecular requirements for the insecticidal activity of the plant peptide pea albumin 1 subunit b (PA1b). J. Biol. Chem. 285, 32689–32694 20660598 10.1074/jbc.M110.147199 PMC2963353

[27] Da Silva P. Strzepa A. Jouvensal L. Rahioui I. Gressent F. Delmas A. F. (2009) A folded and functional synthetic PA1b and interlocked entomotoxin miniprotein. Biopolymers 92, 436–444 19399851 10.1002/bip.21217

[28] Wieczorek H. Cioffi M. Klein U. Harvey W. R. Schweikl H. Wolfersberger M. G. (1990) Isolation of goblet cell apical membrane from tobacco hornworm midgut and purification of its vacuolar-type ATPase. Methods Enzymol. 192, 608–616 2150092 10.1016/0076-6879(90)92098-x

[29] Bockelmann S. Menche D. Rudolph S. Bender T. Grond S. von Zezschwitz P. Muench S. P. Wieczorek H. Huss M. (2010) Archazolid A binds to the equatorial region of the c ring of the vacuolar H^+^-ATPase. J. Biol. Chem. 285, 38304–38314 20884613 10.1074/jbc.M110.137539 PMC2992264

[30] Pauchet Y. Wilkinson P. Chauhan R. Ffrench-Constant R. H. (2010) Diversity of beetle genes encoding novel plant cell wall degrading enzymes. PLoS One 5, e15635 21179425 10.1371/journal.pone.0015635 PMC3003705

[31] Finbow M. E. Goodwin S. F. Meagher L. Lane N. J. Keen J. Findlay J. B. Kaiser K. (1994) Evidence that the 16-kDa proteolipid (subunit *c*) of the vacuolar H^+^-ATPase and ductin from gap junctions are the same polypeptide in *Drosophila* and *Manduca*: molecular cloning of the *Vha16k* gene from *Drosophila*. J. Cell Sci. 107, 1817–1824 7983150 10.1242/jcs.107.7.1817

[32] Walker M. Knight P. Trinick J. (1985) Negative staining of myosin molecules. J. Mol. Biol. 184, 535–542 2413217 10.1016/0022-2836(85)90300-6

[33] Tang G. Peng L. Baldwin P. R. Mann D. S. Jiang W. Rees I. Ludtke S. J. (2007) EMAN2: an extensible image processing suite for electron microscopy. J. Struct. Biol. 157, 38–46 16859925 10.1016/j.jsb.2006.05.009

[34] van Heel M. Harauz G. Orlova E. V. Schmidt R. Schatz M. (1996) A new generation of the IMAGIC image processing system. J. Struct. Biol. 116, 17–24 8742718 10.1006/jsbi.1996.0004

[35] Scheres S. H. Núñez-Ramírez R. Sorzano C. O. Carazo J. M. Marabini R. (2008) Image processing for electron microscopy single-particle analysis using XMIPP. Nat. Protoc. 3, 977–990 18536645 10.1038/nprot.2008.62 PMC2778070

[36] Ludtke S. J. Baldwin P. R. Chiu W. (1999) EMAN: semiautomated software for high-resolution single particle reconstructions. J. Struct. Biol. 128, 82–97 10600563 10.1006/jsbi.1999.4174

[37] Murata T. Yamato I. Kakinuma Y. Leslie A. G. Walker J. E. (2005) Structure of the rotor of the V-type Na^+^-ATPase from *Enterococcus hirae*. Science 308, 654–659 15802565 10.1126/science.1110064

[38] Harrison M. A. Murray J. Powell B. Kim Y. I. Finbow M. E. Findlay J. B. (1999) Helical interactions and membrane disposition of the 16-kDa proteolipid subunit of the vacuolar H^+^-ATPase analyzed by cysteine replacement mutagenesis. J. Biol. Chem. 274, 25461–25470 10464277 10.1074/jbc.274.36.25461

[39] Nelson H. Nelson N. (1990) Disruption of genes encoding subunits of yeast vacuolar H^+^-ATPase causes conditional lethality. Proc. Natl. Acad. Sci. U.S.A. 87, 3503–3507 2139726 10.1073/pnas.87.9.3503 PMC53929

[40] Qin A. Cheng T. S. Pavlos N. J. Lin Z. Dai K. R. Zheng M. H. (2012) V-ATPases in osteoclasts: structure, function and potential inhibitors of bone resorption. Int. J. Biochem. Cell Biol. 44, 1422–1435 22652318 10.1016/j.biocel.2012.05.014

[41] Sennoune S. R. Luo D. F. Martínez-Zaguilán R. (2004) Plasmalemmal vacuolar-type H^+^-ATPase in cancer biology. Cell Biochem. Biophys. 40, 185–206 15054222 10.1385/CBB:40:2:185

[42] Wieczorek H. Brown D. Grinstein S. Ehrenfeld J. Harvey W. R. (1999) Animal plasma membrane energization by proton motive V-ATPases. Bioessays 21, 637–648 10440860 10.1002/(SICI)1521-1878(199908)21:8<637::AID-BIES3>3.0.CO;2-W

[43] Baum J. A. Bogaert T. Clinton W. Heck G. R. Feldmann P. Ilagan O. Johnson S. Plaetinck G. Munyikwa T. Pleau M. Vaughn T. Roberts J. (2007) Control of coleopteran insect pests through RNA interference. Nat. Biotech. 25, 1322–1326 10.1038/nbt1359 17982443

[44] Saliba K. J. Kirk K. (1999) pH regulation in the intracellular malaria parasite, *Plasmodium falciparum*. J. Biol. Chem. 274, 33213–33219 10559194 10.1074/jbc.274.47.33213

[45] Moriyama Y. Hayashi M. Yatsushiro S. Yamamoto A. (2003) Vacuolar proton pumps in malaria parasite cells. J. Bioenerg. Biomembr. 35, 367–375 14635782 10.1023/a:1025785000544

[46] Cociancich S. O. Park S. S. Fidock D. A. Shahabuddin M. (1999) Vesicular ATPase-overexpressing cells determine the distribution of malaria parasite oocysts on the midguts of mosquitoes. J. Biol. Chem. 274, 12650–12655 10212245 10.1074/jbc.274.18.12650

[47] Visentin L. Dodds R. A. Valente M. Misiano P. Bradbeer J. N. Oneta S. Liang X. Gowen M. Farina C. (2000) A selective inhibitor of the osteoclastic V-H^+^-ATPase prevents bone loss in both thyroparathyroidectomized and ovariectomized rats. J. Clin. Invest. 106, 309–318 10903347 10.1172/JCI6145 PMC380241

[48] Benlekbir S. Bueler S. A. Rubinstein J. L. (2012) Structure of the vacuolar-type ATPase from *Saccharomyces cerevisiae* at 11Å resolution. Nat. Struct. Mol. Biol. 19, 1356–1362 23142977 10.1038/nsmb.2422

[49] Merzendorfer H. Huss M. Schmid R. Harvey W. R. Wieczorek H. (1999) A novel insect V-ATPase subunit M9.7 is glycosylated extensively. J. Biol. Chem. 274, 17372–17378 10358099 10.1074/jbc.274.24.17372

[50] Bowman E. J. Graham L. A. Stevens T. H. Bowman B. J. (2004) The bafilomycin/concanamycin binding site in subunit c of the V-ATPases from *Neurospora crassa* and *Saccharomyces cerevisiae*. J. Biol. Chem. 279, 33131–33138 15180988 10.1074/jbc.M404638200

[51] Upadhyay S. K. Chandrashekar K. Thakur N. Verma P. C. Borgio J. F. Singh P. K. Tuli R. (2011) RNA interference for the control of whiteflies (*Bemisia tabaci*) by oral route. J. Biosci. 36, 153–161 21451256 10.1007/s12038-011-9009-1

[52] Páli T. Dixon N. Kee T. P. Marsh D. (2004) Incorporation of the V-ATPase inhibitors concanamycin and indole pentadiene in lipid membranes: spin-label EPR studies. Biochim. Biophys. Acta 1663, 14–18 15157605 10.1016/j.bbamem.2004.03.003

[53] Mizutani K. Yamamoto M. Suzuki K. Yamato I. Kakinuma Y. Shirouzu M. Walker J. E. Yokoyama S. Iwata S. Murata T. (2011) Structure of the rotor ring modified with *N*,*N*′-dicyclohexylcarbodiimide of the Na^+^-transporting vacuolar ATPase. Proc. Natl. Acad. Sci. U.S.A. 108, 13474–13479 21813759 10.1073/pnas.1103287108 PMC3158168

