# Supplementary material for: PA1b Inhibitor Binding to Subunits c and e of the Vacuolar ATPase Reveals Its Insecticidal Mechanism
Source: J Biol Chem. 2014 May 2;289(23):16399–408. doi: 10.1074/jbc.M113.541250 (PMC4047407; doi:10.1074/jbc.M113.541250)
Supplement: Supplemental Data [file supp_289_23_16399__index.html]

PA1b Inhibitor Binding to Subunits c and e of the Vacuolar ATPase Reveals its Insecticidal Mechanism — PA1b Inhibitor Binding to Subunits c and e of the Vacuolar ATPase Reveals Its Insecticidal Mechanism — Characterization of PA1b Binding — Supplemental Data 

# PA1b Inhibitor Binding to Subunits *c* and *e* of the Vacuolar ATPase Reveals Its Insecticidal Mechanism

## Supplemental Data

**Files in this Data Supplement:**

- Supplemental Figure 1 (.jpg, 437 KB) - Negative stain classes for samples A V-ATPase; B V-ATPase with streptavidin-HRP; C V-ATPase with 5% ethanol (control for the PA1b solvent); D V-ATPase with biotin-PA1b; E V-ATPase with simultaneously added biotin-PA1b and streptavidin-HRP and F V-ATPase with biotin-PA1b/streptavidin-HRP complex pre-formed by overnight conjugation. Those classes showing PA1b binding are highlighted by a star. The scale bar represents 15nm.
