# Supplementary figures and images for: PA1b Inhibitor Binding to Subunits c and e of the Vacuolar ATPase Reveals Its Insecticidal Mechanism
Source: J Biol Chem. 2014 May 2;289(23):16399–408. doi: 10.1074/jbc.M113.541250 (PMC4047407; doi:10.1074/jbc.M113.541250)

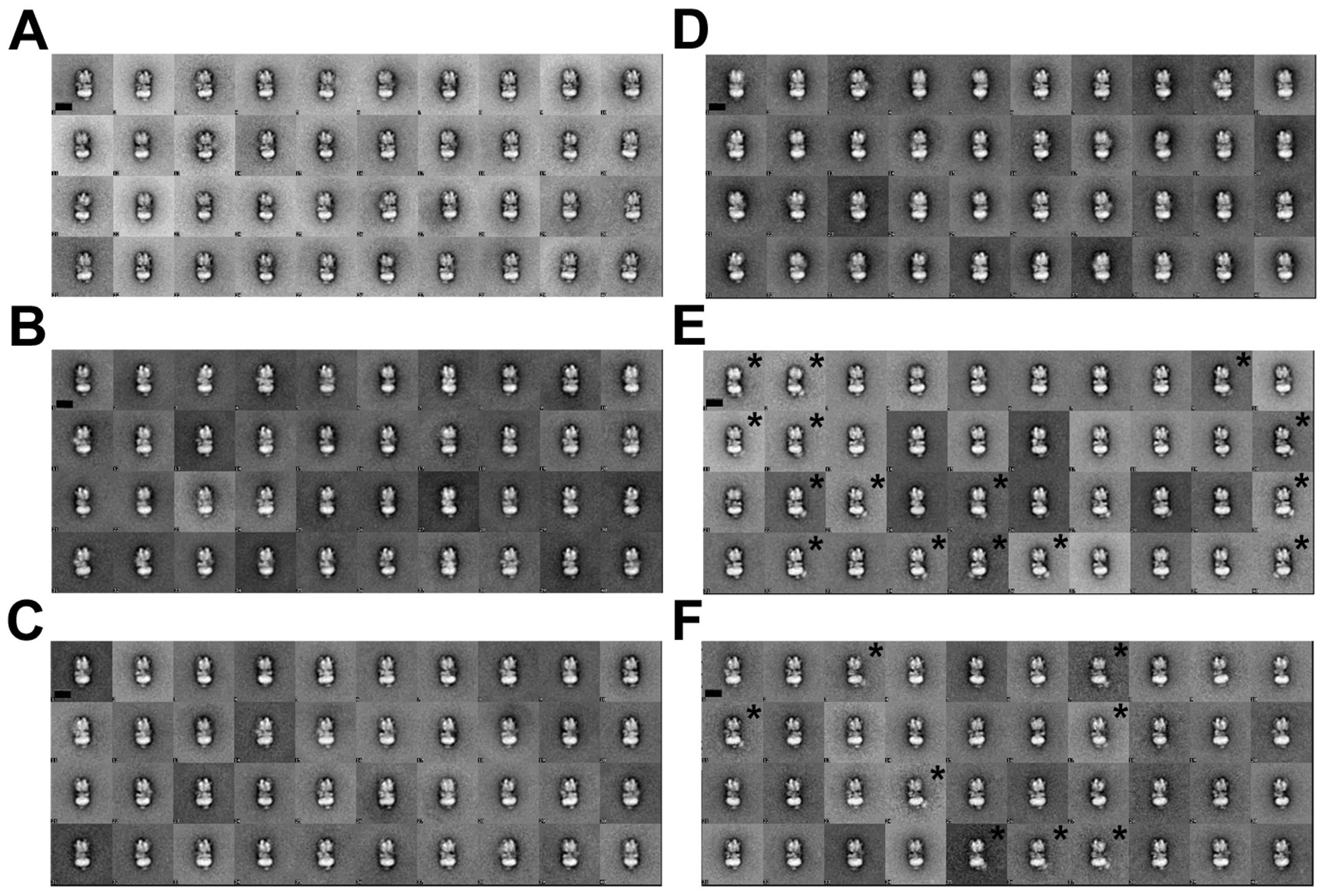

Supplement: Supplemental Data [file supp_M113.541250_jbc.M113.541250-1.jpg]
